# Patient Experiences With Hand Surgery in the Office Versus Ambulatory Surgery Center

**DOI:** 10.7759/cureus.43763

**Published:** 2023-08-19

**Authors:** Brandon W Knopp, Jared Kushner, Emma Eng, Jake Goguen, Ehsan Esmaeili

**Affiliations:** 1 Endocrinology, Florida Atlantic University Charles E. Schmidt College of Medicine, Boca Raton, USA; 2 Orthopedic Surgery, Florida Atlantic University Charles E. Schmidt College of Medicine, Boca Raton, USA; 3 Orthopedic Surgery, South Florida Hand and Orthopaedic Center, Boca Raton, USA

**Keywords:** surgical setting, plastic and reconstructive surgery, orthopedic surgery, walant, hand surgery, patient satisfaction

## Abstract

Background

In hand surgery, physicians are working to improve patient satisfaction by offering several minor procedures in the physician’s office via the Wide-Awake Local Anesthesia No Tourniquet (WALANT) method. This study investigates the degree of patient satisfaction, out-of-pocket costs, peri- and postoperative pain, convenience, and comfort experienced with in-office hand procedures compared to ambulatory surgery center (ASC) procedures.

Methods

A 10-question survey consisting of a 10-point Likert scale of agreement and numerical questions was administered to patients treated with minor hand operations in the office and ASC settings in Florida, USA. The surgical procedures included are bony reconstruction, percutaneous pinning, open reduction internal fixation, closed fracture reduction, mass removal, endoscopic carpal tunnel release, Dupuytren's release/tendon repair, and trigger finger release. Procedures and patient demographics were assessed via chart review. Independent samples t-test was used to determine statistical associations with significance defined as p < 0.05.

Results

Patients reported a strong level of agreement in response to questions 1-3 and 6-8, indicating a high degree of convenience, comfort, and overall satisfaction with both in-office and ASC procedures. Positive metrics gauged in questions 1-3 and 6-8 averaged 9.64 ± 0.14 in the office setting and 9.62 ± 0.16 in the ASC setting. Questions 4 and 5 averaged 2.74 ± 0.29 in the office setting and 2.84 ± 4.12 in the ASC setting, indicating mild disagreement that the surgery or recovery period was painful. In-office patients reported taking 0.91 ± 2.80 days off work and ASC patients reported taking 12.43 ± 22.51 days off work following surgery (p = 0.0039). Respondents reported an out-of-pocket cost averaging $348 ± $943 in the office setting and $574 ± $1262 in the ASC setting, depending on insurance coverage (p = 0.3019).

Conclusions

Though costs and time off of work differed between the two groups due to the different procedures in either setting, patient satisfaction metrics were comparable. While patient satisfaction depends on the operating physician, these results demonstrate that patients treated in-office and in an ASC have similar levels of approval with their hand surgery care.

## Introduction

Patient satisfaction is an important quality outcome indicator for clinical care. It positively correlates with a higher quality of care and has several implications for patients and healthcare providers. Patient satisfaction benefits include improved patient retention, greater profitability, greater satisfaction for staff and physicians, and reduced incidence of malpractice suits. Patient satisfaction may also indicate the quality of care [1-6].

In hand surgery, physicians are working to improve patient satisfaction by offering in-office procedures to patients for minor surgeries including carpal tunnel release, trigger finger release, needle aponeurotomy, fracture reduction, and mass removal. In-office procedures are done in procedural rooms (PRs) adapted for minor procedures via the Wide-Awake Local Anesthesia No Tourniquet (WALANT) method of local anesthesia and hemostasis [7,8]. This growing trend in hand surgery can improve patient satisfaction by offering safe, efficient care delivery in the office. Several potential benefits of in-office PR procedures are shorter wait times for surgery scheduling, greater interaction with patients during WALANT procedures, lower medical payments, and fewer return visits [8-17]. According to a study conducted by Rhee et al. in 2017 which included 67 patients undergoing carpal tunnel release and A1 pulley release surgeries in-office, 94% of those patients would choose WALANT again [18].

There is ample evidence to support the benefits of in-office procedures, however, more data is required to determine patient satisfaction following in-office procedures compared to procedures done in ambulatory surgery centers (ASCs) or hospitals. This study seeks to perform a unit analysis of patients who underwent several common hand procedures in the office and ASC settings to determine if the procedural setting impacts patient satisfaction. In addition to evaluating the impact of procedural settings on patient satisfaction, we seek to understand the extent to which patients are satisfied with the care process. This includes overall satisfaction, convenience in scheduling, comfort in the procedural setting, peri- and postoperative pain, time taken off of work, and out-of-pocket costs. Evaluating patient satisfaction between these settings will inform hand surgeons how to best serve their patients and lead to more patient-centered care delivery.

## Materials and methods

A retrospective review of patients who underwent minor hand operations in the office PR or ASC between December 2020 and December 2021 in Florida, USA, was performed. All patients were treated by a single orthopedic hand surgeon. The surgical procedures included are the hook of hamate excision, digital amputation revision, percutaneous pinning, open reduction internal fixation, closed fracture reduction, mass removal, endoscopic carpal tunnel release, Dupuytren’s release/tendon repair, and trigger finger release. All patients were scheduled for a post-operative follow-up per standard practice of the operating surgeon. Inclusion criteria were: (1) underwent one of the aforementioned procedures in the office PR or ASC, (2) completed the postoperative phone survey, and (3) attended a postoperative follow-up visit. There were no exclusion criteria.

**Figure 1:**
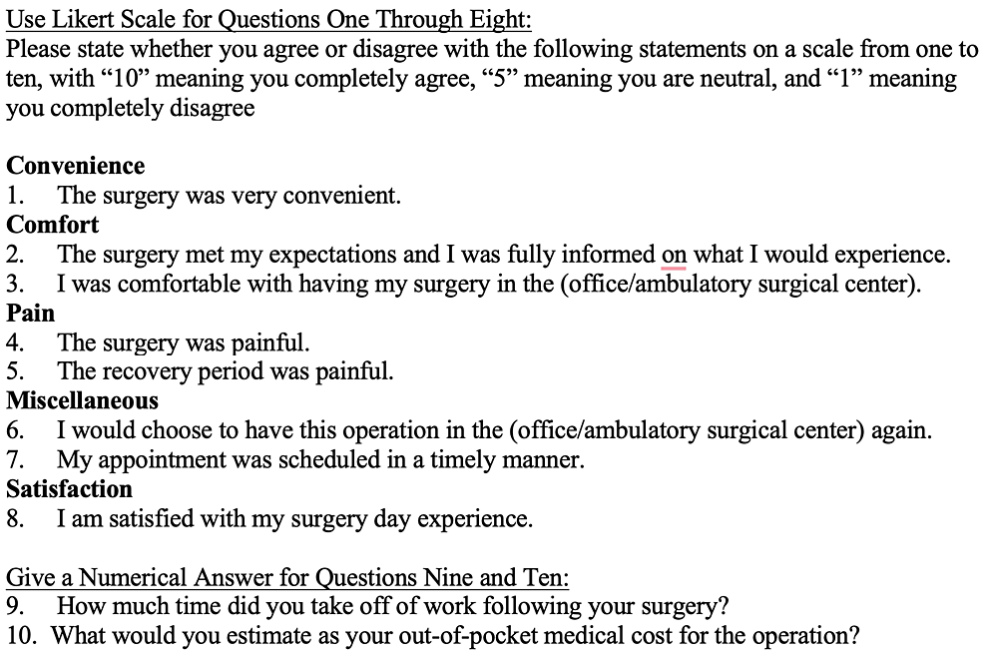
Patient satisfaction survey

A query identified 437 patients who underwent minor hand operations, including 141 patients treated in an office procedure room and 296 patients treated in an ASC. All patients were attempted to be contacted to administer a 10-question survey consisting of a 10-point Likert scale of agreement and numerical questions. The survey administered to participants is shown in Figure 1.

Questions investigated aspects of the surgical experience including convenience, comfort, pain, overall satisfaction, time taken off work, and out-of-pocket cost. Questions 1-3 and 7 asked about patient comfort and convenience in the surgical setting. Questions 4 and 5 asked about patient pain in the operative and postoperative periods. Questions 6 and 8 asked about overall patient satisfaction. Question 9 asked about the time taken off of work following the surgery and question 10 asked about out-of-pocket costs for the operation.

Patient demographic data, surgical history, and contact information were obtained via electronic health records (EHRs). Trained researchers administered all surveys via telephone interview. All participants were informed of the purpose of the study and gave informed consent to be included in the study at the beginning of the telephone interview. Patients who did not answer the first call were called one additional time on a different day. An independent samples t-test was used to evaluate continuous variables obtained via chart review and survey responses. Significance was defined as p < 0.05.

## Results

A total of 151 patients treated in the office setting (58) and ASC setting (93) were reached via phone call and consented to complete the survey (35% response rate). There were 59 male and 92 female respondents with an average age of 63.60 ± 15.47 years and no significant age or gender differences between the in-office and ASC groups. More invasive operations, such as endoscopic carpal tunnel release and fracture reduction were more frequently performed in the ASC setting (Table 1).

**Table 1:** Procedures done in the ASC and office procedure room ASC, Ambulatory Surgery Center

	ASC Procedures	In-Office Procedures
Bony Reconstruction	7	0
Percutaneous Pinning	6	0
Open Reduction Internal Fixation	18	0
Closed Fracture Reduction	0	2
Mass Removal	9	10
Endoscopic Carpal Tunnel Release	35	1
Dupuytren’s Release or Tendon Repair	4	12
Trigger Finger Release	19	33
Multiple Procedures Done During Surgery	6	0

Survey responses for in-office patients are seen in Table 2 and for ASC patients in Table 3. Survey questions 1-8 are answered on a scale from 1 to 10, with an answer of 10 indicating the highest level of agreement. Survey responses indicate strong agreement with the positive metrics in questions 1-3 and 6-8 for in-office and ASC patients. Questions 4 and 5 indicate mild disagreement that the surgery or recovery period was “painful” in the office or ASC setting. In response to question 6, all but six patients (4.0%) in both groups responded with answers of seven or above, indicating a moderate to strong intention of having future hand surgeries in the same setting if available.

**Table 2:** Survey responses for in-office patients

	Question 1: Convenience	Question 2: Expectations were Met	Question 3: Comfort	Question 4: Surgery was Painful	Question 5: Recovery was Painful	Question 6: Would Choose Setting Again	Question 7: Timely Scheduling	Question 8: Patient Satisfaction
Average	9.69	9.38	9.79	2.53	2.95	9.62	9.64	9.71
Standard Deviation	1.03	1.47	0.81	2.27	2.37	1.41	1.00	1.08
Count	58	58	58	58	58	58	58	58

**Table 3:** Survey responses for ASC patients ASC, Ambulatory Surgery Center

	Question 1: Convenience	Question 2: Expectations were Met	Question 3: Comfort	Question 4: Surgery was Painful	Question 5: Recovery was Painful	Question 6: Would Choose Setting Again	Question 7: Timely Scheduling	Question 8: Patient Satisfaction
Average	9.47	9.42	9.81	2.00	3.69	9.57	9.81	9.66
Standard Deviation	1.46	1.65	0.80	2.03	2.80	1.62	1.01	1.57
Count	93	93	93	93	93	93	93	93

39.6% of participants were retired at the time of surgery, with no significant difference between the groups (p = 0.7225). Among employed respondents, those treated in the office reported taking 0.91 ± 2.80 days off work, while patients treated in the ASC reported taking 12.43 ± 22.51 days off work (p = 0.0039). Respondents also reported an out-of-pocket cost averaging $348 ± $943 in the office setting and $574 ± $1,262 in the ASC setting with large differences depending on insurance or Medicare coverage (p = 0.3019). Aside from time taken off work to recover, there were no significant differences in survey responses or patient characteristics between the two surgical settings.

## Discussion

The prevalence of in-office hand surgery has been increasing over recent years [14]. A hybrid model of care with minor procedures offered more frequently in the office setting and more invasive operations solely done in the ASC or hospital setting is becoming more prevalent. This model allows a more convenient mode of care delivery and increases the availability of ASC and hospital operating rooms by conducting more minor procedures in the office setting. At this early stage, offerings depend primarily on provider comfort with operating in the office setting for select procedures and individual surgeon assessments of the level of support services required for each operation [15]. This evaluation depends on patient characteristics, operational complexity, and the need for general anesthesia or regional nerve blocks.

The precise indications which may deem an operation appropriate in the office setting for a given patient have yet to be identified. However, our survey results suggest that further investigation of in-office surgery safety and patient satisfaction would benefit many practicing physicians [18]. While the elevated level of support offered in the ASC is required to safely perform more invasive surgeries, operating in the office has multiple benefits for the provider and patient [3,4,12,15,16]. First, the convenience of operating in the office setting allows surgeons to see more patients without leaving the office and minimizes unnecessary anesthesia use [7,9]. This enables physicians to see patients more efficiently with shorter wait times [15]. The office setting is particularly appealing in regions with a shortage of operating room availability as it can decrease demand for ASC and hospital operating rooms without compromising patient safety or satisfaction.

As exemplified by the differences in recovery time between patients treated in the office (0.91 ± 2.80 days off work) and ASC (12.43 ± 22.51 days off work), less invasive operations are more frequently performed in an office PR (Table 1). These less invasive hand surgeries are optimally done in the office for many patients as they require fewer supportive resources such as general anesthesia, regional nerve blocks, and support staff. In-office surgeries can be done with local anesthesia, antiseptic skin preparation, standard surgical equipment with a sterilized field, and an assistant. The easier accessibility of in-office surgeries is particularly beneficial for operations such as trigger finger releases which more frequently require future surgeries to treat disease recurrence.

Though statistically insignificant, there is a difference in patient out-of-pocket costs for surgery between the two settings ($348 ± $943 in-office compared to $574 ± $1262 in the ASC setting). Potentially due to a type II error, this difference merits further investigation with consideration of insurance coverage and surgeries undergone. However, despite the differences in the types of surgeries performed in each setting, there was no significant difference in respondent ratings between the two surgical settings. Positive metrics regarding patient convenience, comfort, timely scheduling, overall satisfaction, and willingness to have a procedure in the same setting again (Questions 1-3 and 6-8) were nearly identical between in-office and ASC surgeries. Likewise, negative metrics regarding operative and post-operative pain had no significant difference between the two settings (Questions 5 and 6), with responses indicating a mild amount of pain experienced.

Overall, the respondents reported positive ratings for in-office and ASC surgeries with no significant differences in survey responses, aside from a greater amount of time being taken off of work following surgeries in an ASC. Patients in both settings reported a small degree of pain in the operative and post-operative period (Questions 4 and 5), a high degree of comfort and convenience (Questions 1-3, 7, and 9), and a high degree of satisfaction (Questions 6 and 8). Though out-of-pocket costs and time taken off of work differed between the two groups due to the different procedures done in each setting, patient satisfaction metrics were comparable between the two groups. While the operating physician influences patient satisfaction, these results demonstrate that patients have similar levels of approval with their hand surgery care via in-office and ASC surgeries.

An opportunity for further research in this area would be to compare the costs for the providers between in-office procedures and ASC procedures. A previous study found that carpal tunnel surgeries performed in operating rooms were significantly more costly than those performed in clinic PRs [15]. Future research can also expand upon these findings by comparing the costs of a broader range of procedures between the two settings. Limitations to this study include the sample size and the method of surveying patients via phone calls.

## Conclusions

Our results support the use of in-office procedures for minor hand surgeries from a patient perspective and indicate a nearly universal intent to repeat any future hand operations in the office setting if given the option. Future research areas may further explore patient opinions on hand surgeries in varied settings for specific operations and investigate cost comparisons between surgeries based on the surgical setting. Patients treated in-office PRs overwhelmingly endorsed having their procedures done in the office, highlighting the potential benefit for patients and providers of operating in the office for select procedures and patients.
